# Symbiotic relationship between filamentous algae (*Halomicronema* sp.) and extracellular polymeric substance-producing algae (*Chlamydomonas* sp.) through biomimetic simulation of natural algal mats

**DOI:** 10.3389/fmicb.2023.1176069

**Published:** 2023-05-24

**Authors:** Ha Eun Lee, Jun Ho Lee, Seung Moon Park, Dae Geun Kim

**Affiliations:** ^1^LED Agri-bio Fusion Technology Research Center, Jeonbuk National University, Iksan-si, Jeollabuk-do, Republic of Korea; ^2^Department of Agricultural Chemistry, Jeonbuk National University, Jeonju-si, Jeollabuk-do, Republic of Korea; ^3^Department of Bioenvironmental Chemistry, Jeonbuk National University, Jeonju-si, Jeollabuk-do, Republic of Korea

**Keywords:** extracellular polymeric substance, filamentous algae, viscous algae, biomass, biomimetic algal mat

## Abstract

To lower the cost of biomass harvesting, the growth of natural biofilm is considered to be an optimal alternative to microalgae aggregation. This study investigated algal mats that naturally agglomerate into a lump and float on water surfaces. *Halomicronema* sp., a filamentous cyanobacterium with high cell aggregation and adhesion to substrates, and *Chlamydomonas* sp., which grows rapidly and produces high extracellular polymeric substances (EPS) in certain environments, are the main microalgae that make up selected mats through next-generation sequencing analysis. These two species play a major role in the formation of solid mats, and showed a symbiotic relationship as the medium and nutritional source, particularly owing to the large amount of EPS formed by the reaction between EPS and calcium ions through zeta potential and Fourier-transform infrared spectroscopy analysis. This led to the formation of an ecological biomimetic algal mat (BAM) that mimics the natural algal mat system, and this is a way to reduce costs in the biomass production process as there is no separate treatment process for harvesting.

## 1. Introduction

Against the backdrop of the ongoing depletion of biological resources, microalgal biomass is a sustainable solution because it can be produced without competing with food resources and it is utilized in various fields such as bioenergy, bioplastics, and animal feed (Huang et al., 2022). In particular, common and natural mat-type microalgae mixtures have various ecological functions, thus necessitating the elucidation of their symbiotic relationships and their increased availability as future resources.

Microalgal biofilm is a living structure composed of protozoa, bacteria, larvae, and microalgae, with various functions owing to the interactions between multiple species (Katarzyna et al., 2015). Biofilms are also microbial consortia embedded in extracellular polymeric materials (EPS) and form complex structures on the solid surface (bottom layer) (Mantzorou and Ververidis, 2019). Biofilm formation occurs when microorganisms colonize surfaces, and as the colonies grow they become more organized within the extracellular matrix, and eventually some organisms disperse to colonize different locations on the surface (Polizzi et al., 2017).

Microbial mats consist of various basic biofunctional groups, including cyanobacteria, anoxygenic photosynthetic bacteria (typically represented by non-sulfur, green Chloroflexi bacteria), aerobic heterotrophs and anaerobes, sulfate-reducing bacteria (SRB), sulfur oxidizing bacteria, and methanogenic archaea (Van Gemerden, 1993; Prieto-Barajas et al., 2018). Jungblut et al. (2016) confirmed that cyanobacteria (such as *Leptolyngbya* and *Phormidium*) and proteobacteria were the major taxa present in the microbial mat of a lake. In addition, green algae and diatoms are eukaryotes often found in microbial mats (Bolhuis et al., 2014), with cyanobacteria being significant members and the primary producers of phototrophic mats (Prieto-Barajas et al., 2018).

The extracellular matrix plays an important role in forming microalgal biofilms because it acts as a barrier by protecting the entire colony (d’Abzac et al., 2010). Moreover, extracellular polymeric substances (EPS) released during the life cycle play a major role in the formation of microalgae biofilm (d’Abzac et al., 2010). Because they form a matrix of anionic molecules, EPS have great metal binding potential (Decho and Gutierrez, 2017). Moreover, EPS significantly contribute to the stability of microalgae biomass because their composition and properties play a role in maintaining the structure and strength of microbial aggregates, thereby significantly contributing to the stability of microalgae biomass (More et al., 2014). In addition, granulated algae cells facilitate harvesting through their interaction with bubbles. The zeta potential of the cell surface and EPS is a major influencing factor of the interaction between algal cells and bubbles (Matho et al., 2019). Cells are negatively charged, and environmental conditions such as pH adjustment and the use of cationic surfactants are required to facilitate harvesting via bubble-algae interaction in water, thereby incurring additional costs.

Generally, harvesting microalga cultures is expensive, and biofilm-type culture methods are widely applied to address this problem; however, further research on aspects of its production, such as efficient harvesting and unit price reduction is required (Witarsa et al., 2020; Zhao et al., 2021). Watari et al. (2021) confirmed that mixing microalgae and bacteria in biofilm maximizes carbon absorption and biomass production, and reduces costs (Wicker et al., 2022). They also found that nitrifying bacteria provide nitrogen for microalgae growth through simultaneous cultivation with microalgae in biofilm. These species mixtures can induce an increase in the concentration and lipid content of biomass in biofilms (Cho et al., 2015), as well as the formation of biofilms with porous microstructures owing to pH changes in the two microalgae. In studies aimed at biofilm improvement, Wicker et al. (2022) explained the importance of biological interactions. In particular, the coexistence of two or more biofilms in close proximity at the picosphere level can create very positive synergy, and the biomembrane culture system that mimics it is economical. This phenomenon can be effectively achieved through natural biofilm simulation.

In this study, *Halomicronema* sp. and *Chlamydomonas* sp., the dominant species that make up a solid microalgae mat found in a lake in Chungcheongnam-do, Korea, were selected as samples, and the solidity and buoyancy mechanism of natural microalgae mats owing to the interaction of the two species were examined. The changes in EPS and the adsorption of Ca^2+^, which play an important role in the aggregation of microalgae and the formation of colonies of the two species, were tracked to explore the conditions of algae mats that can coagulate for a long time without additional equipment or chemicals. By replicating the interactions between different types of algae, these biomimetic simulations can provide insight into the conditions under which naturally robust clusters are formed and maintained for a long time without cell detachment. This is beneficial to the study of complex ecosystem responses. In addition, the study results introduce a new culture method that could potentially produce biomass at a low cost based on the interaction of two species and without the facilities or attachment materials required for existing biofilm systems, along with potential applications.

## 2. Materials and methods

### 2.1. Selection of strains and cultivation

Microalgae strains were used by selecting *Halomicronema* sp. of Cyanobacteria and *Chlamydomonas* sp. of Chlorophyceae, the dominant species in the next-generation sequencing analysis of microalgae mats collected from a lake in Chungcheongnam-do, Korea. Optimal *Haematococcus* medium (OHM) was used for strain cultures (Fábregas et al., 2000). It was sterilized at 121°C for 15 min and then cooled. For strain growth and culture mixing, aeration was performed at 1 vvm, and air was injected through a 0.45 μm syringe filter (Sartorius, Gottingen, Germany) to minimize contamination. To determine the optimal growth conditions for each strain, temperature conditions were maintained between 20°C and 30°C, which is the general culture temperature range of green microalgae and cyanobacteria, and the light conditions were approximately 120 μmol m^−2^ s^−1^ (HL, High Light) and 60 μmol m^−2^ s^−1^ (LL, Low Light), respectively.

### 2.2. Experimental setup

#### 2.2.1. Pure and mixed cultures

For pure culture, 500 mL of medium was used in a 1 L round flask, and the initial concentration of the strain was set to 0.2 g L^−1^. After incubation for 7 days, the whole sample was harvested to measure the dry weight of the cells. Optical density (OD) was measured at a wavelength of 660 nm to confirm the aggregation and growth of each algae. Soluble EPS (s-EPS) and bound EPS (b-EPS) on the first and last days of culture were extracted and quantified to measure EPS production during culture.

In the mixed culture, 100 mL of medium was used in a 250 mL round flask, and *Halomicronema* sp. and *Chlamydomonas* sp. were set to an initial concentration of 0.6 g L^−1^ respectively, mixed, and then cultured. After incubation for 7 days, the growth of the mixed cluster was calculated by harvesting the entire cluster for cell dry weight measurement. OD was measured at a wavelength of 660 nm to confirm the change in aggregation and dispersion between the two algae by mixed culture. After mixing the two algae, s-EPS was measured when the culture was properly stabilized and on the last day. In b-EPS, 0.1 g was removed from the mixed algae mat to measure the change in content.

#### 2.2.2. S-EPS consumption by *Halomicronema* sp.

In order to verify the s-EPS consumption of *Halomicronema* sp., the s-EPS of *Chlamydomonas* sp. was extracted and prepared as s-EPS solution, four groups of experimental biomass growth were set up, such as (1) s-EPS solution, (2) s-EPS solution + *Halomicronema* sp. (3) Nutrients + *Halomicronema* sp. (4) s-EPS solution + Nutrients + *Halomicronema* sp. For each experimental group, 0.2 g L^−1^ of *Halomicronema* sp. was used. To confirm the s-EPS consumption of *Halomicronema* sp., s-EPS at a concentration of 400 mg L^−1^ was supplied on the first day of the experiment and s-EPS was measured once daily. Total nitrogen and total phosphorus were analyzed to confirm the amount of nutrients removed by *Halomicronema* sp.

### 2.3. Analytical method

#### 2.3.1. Microalgae growth

The initial concentration of pure and mixed culture strain was set to 0.2 and 1.2 g L^−1^, respectively. Cells were filtered through a 1.2 μm GF/C glass fiber filter paper (Whatman, Dassel, Germany) and dried at 70°C for 2 h in a drying oven (VS–4150ND, Vision Scientific Co. Ltd., Korea). The dry cell weight (DCW) was calculated by measuring the weight of the dried filter paper using an analytical electronic balance (Shimadzu Corporation, Japan). Absorbance was measured at 660 nm, the maximum absorption wavelength of chlorophyll a, using a UV/Vis spectrophotometer (T60, PG instruments, United Kingdom).

#### 2.3.2. Next-generation sequencing analysis

Herein, mat-type microalgal clusters collected from a lake in Chungcheongnam-do, Korea, were used for the study of microalgae. An Illumina MiSeq platform (Macrogen Inc., Seoul, Korea) was used to obtain the base sequence of the 16S ribosomal RNA (rRNA) gene and the 18S rRNA gene V4-V5 region of the collected microalgae mats. Sequencing was performed using the Quantitative Insights into Microbiology (QIIME) program (Caporaso et al., 2010). Based on the sequence information, microalgae classification by 16S rRNA genes was based on the NCBI_16S_ribosomal_RNA_20201201 (BLAST) database and that by 18S rRNA genes on the NCBI_NT_20202 (BLAST) database. One operational taxonomic unit (OTU) was classified by clustering more than 97% of similar sequences. Afterwards, two species that appeared to be dominant were selected from the NGS analysis results of the microalgae clusters and used as test strains after pure culture isolation.

#### 2.3.3. Extraction and determination of extracellular polymeric substances

Extracted EPS were divided into s-EPS and b-EPS (Kim et al., 2021). For the extraction of s-EPS, the culture sample was filtered using 1.2 μm GF/C glass fiber filter paper (Whatman, Dassel, Germany). For the extraction of b-EPS, the culture sample was centrifuged at 13,500 rpm for 10 min and then the supernatant was discarded. A 0.9% NaCl solution of the same volume as the initial sample amount was added, vortexed, and then heat treated at 80°C for 10 min to separate cells from EPS. After centrifugation at 13,500 rpm for 10 min, the supernatant was filtered using a GF/C glass fiber filter paper to extract b-EPS. The EPS content was expressed as the content of polysaccharides and proteins, which are the main components. Polysaccharide analysis was quantified using glucose as a standard according to the phenol-sulfate method of Dubois et al. (1951). The extracted EPS sample (0.5 mL) is added to 0.5 mL of a 5% phenol solution and 1 mL of 95% sulfuric acid, and then shake the mixture vigorously. After cooling to room temperature (25°C) for 10 min, absorbance was measured at 490 nm. Protein analysis was performed using the Lowry method (Classics Lowry et al., 1951), and bovine serum albumin (BSA) was quantified as a standard. First, an alkaline solution is prepared by dissolving 4 g of sodium carbonate in 100 mL of 0.8% sodium hydroxide (Lowry A solution). Two percent copper sulfate and 4% sodium tartarate are mixed in a 1:1 ratio to create the Lowry B solution. The Lowry A solution is then mixed with the Lowry B solution in a 50:1 ratio. The extracted EPS sample (0.5 mL) is added to 2.5 mL of alkaline copper solution and mixed vigorously for 1 min. Next, 250 μL of phenol reagent (folin solution) was added and mixed and after 30 min, the absorbance was measured at 750 nm.

#### 2.3.4. Total nitrogen (TN) and total phosphorus (TP) analysis

TN and TP were measured using commercially available kits (HS-TN-L and HS-TP-L, respectively) (Humas, Daejeon, South Korea). For TN, 6 mL of potassium persulfate alkalized with 5% NaOH was added to 2 mL of the sample and shaken 10 times (Georghiou and Ho, 1989). Thereafter, the mixture was heated at 120°C for 30 min to decompose organic matter in the sample and oxidize the nitrate ions, and then cooled with water to room temperature. For color development of the pretreated sample, chromotropic acid (4, 5-dihydroxy-2, 7-naphthalene disulfonic acid) was added to the mixture. Twelve milliliters of 10% sulfuric acid was added to maintain the acidity of the sample. For TP, 1 mL of potassium persulfate was added to 5 mL of the sample and heated at 120°C for 30 min to convert all phosphorus compounds in the sample to phosphate (Nagul et al., 2015). Thereafter, the mixture was cooled with water to room temperature and mixed with 2 mL of ammonium molybdate-ascorbic acid solution for color development. TN and TP were quantified using absorbance at 415 and 880 nm, respectively, by a UV/Vis spectrophotometer (HS-3700, Humas, Korea).

#### 2.3.5. Zeta potential

The zeta potentials of algae and EPS were determined using a Zetasizer (NANO ZS, Malvern Instrument Ltd., United Kingdom). The samples used in the experiment were freeze-dried with cells of pure cultured *Halomicronema* sp. and *Chlamydomonas* sp., BAM mats formed by mixing and culturing, and EPS extracted from *Chlamydomonas* sp. Thereafter, the dried sample was crushed using a mortar, dispersed at a concentration of ≤1%, and then placed in a cuvette for measurement.

#### 2.3.6. Fourier-transform infrared spectroscopy (FTIR)

Spectra were obtained using FTIR (FT/IR-4100, Jasco, Japan) to identify the surface functional groups of each biomass. FT-IR measurement was performed on samples constituting cells of *Halomicronema* sp. and *Chlamydomonas* sp. pure cultured for 7 days, BAM mats formed by mixing and culturing, and EPS extracted from *Chlamydomonas* sp. After freeze-drying, samples changed with a mortar were used. The sample was placed on a crystal and pressure was applied using attenuated total reflection (ATR) to connect the sample with the contact surface. Absorbance spectra were collected between 4,000 and 650 cm^−1^ at a spectral resolution of 4 cm^−1^, and 100 scans were summed up and averaged.

#### 2.3.7. Microscopy observation

Cells and EPS were placed on a glass slide, incubated at room temperature for 30 min, and observed under a biological microscope (KB-500, Korealabtech co., Korea). Then, 0.03% Alcian blue dye was mixed with 0.06% acetic acid and 1 mL of this mixture was applied to a glass slide (Vergnes et al., 2019). The surface of the glass slide was soaked in distilled water for 15 min to remove excess staining reagent.

The surface binding morphological characteristics of Ca^2+^ were analyzed using field emission scanning electron microscopy (FE-SEM; Supra 40 Vp, Carl Zeiss, Germany). After lyophilizing the sample for SEM scanning, the dried sample was attached to a specimen holder with double-sided carbon tape and coated with platinum using an ion sputter coater (EM ACE 600, Leica, Germany) (Yehuda et al., 2021). Additionally, Ca^2+^ on cell surfaces and EPS were quantitatively calculated using an energy-dispersive X-ray spectroscopy (EDS) detector attached to the FE-SEM.

### 2.4. Calculation

#### 2.4.1. Specific growth rate

The specific growth rate (μ) of algal biomass was calculated using the following equation:

(1) $$\mu \left(d^{-1}\right)=3.322\times \frac{\log_{10}\left(\frac{N_{1}}{N_{2}}\right)}{t_{1}-t_{2}}\;$$

where *μ*, d^−1^ is the specific growth rate, *N*_1_ is the biomass concentration on the last day, *N*_2_ is the biomass concentration on the first day, *t*_1_ is the last day, and *t*_2_ is the first day.

#### 2.4.2. Buoyancy test

Microalgae buoyancy was calculated using the following equation.

(2) $$F_{B}=-gV\left(\rho_{a}-\rho_{w}\right)\;$$

where g is gravitational acceleration (9.81 m s^−2^), *V* is the volume of the algae, ρ_a_ is the density of the algae, and ρ_w_ is the density of the medium. The volume of algae was determined by measuring the additional volume of water when the entire algae was submerged in a graduated cylinder. Algal density was calculated by dividing the mass by the volume, and the mass was calculated by measuring the DCW. The density of the medium was calculated to be 1 g cm^−3^, which is the commonly considered density of water.

### 2.5. Statistics analysis

The analysis values were expressed as mean ± standard deviation, and a one-way analysis of variance (ANOVA) test was conducted using the Pasw statistics 18 (SPSS Inc., Version 18.0) program. The significance level between means was determined by Duncan’s *post hoc* test at *p* < 0.05.

## 3. Results and discussion

### 3.1. Eukaryotic and prokaryotic microalgae diversity evaluation of wild microalgal mat for ecological mimicry

**Figure 1:**
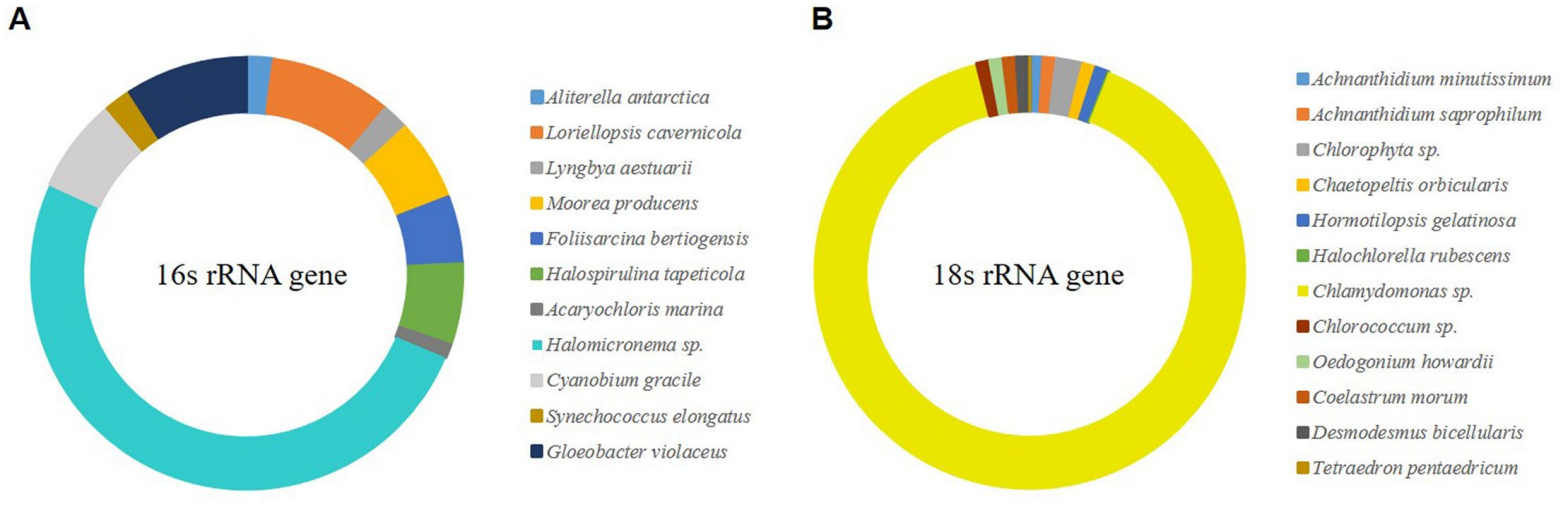
NGS analysis of wild-type microalgal mat (>0.1% relative abundance). **(A)** The V3–V4 region of 16S rRNA gene. **(B)** The V4–V5 region of 18S rRNA gene.

Microalgal biofilms comprise a mixture of various life forms with unique roles (Scognamiglio et al., 2021). The collected biofilm has unique robustness, which may be influenced by the constituent organisms that make up it; therefore, sequencing and OTU clustering were conducted through NGS. Species were constructed by selecting OTUs with a frequency of ≥1%, and as a result, 10 OTUs were selected for eukaryotic microalgae and 11 OTUs for prokaryotic microalgae (Figure 1). OTU, which showed 91% of the selected eukaryotic microalgae, was confirmed to be *Chlamydomonas* sp. of green algae, and *Halomicronema* sp., which exhibited 50% cyanobacteria. *Chlamydomonas* sp. produces various EPS components (Allard and Tazi, 1992), and *Halomicronema* sp. forms various microalgal mats (Zupo et al., 2019). Therefore, both species could contribute significantly to the composition of microalgal biofilms in terms of relative abundance and functional aspects. Through pure culture isolation, the differences in characteristics between pure and mixed cultures were compared.

### 3.2. Characteristics of pure cultures of *Halomicronema* sp. and *Chlamydomonas* sp. under light and temperature conditions

#### 3.2.1. Growth biomass and optical density

**Figure 2:**
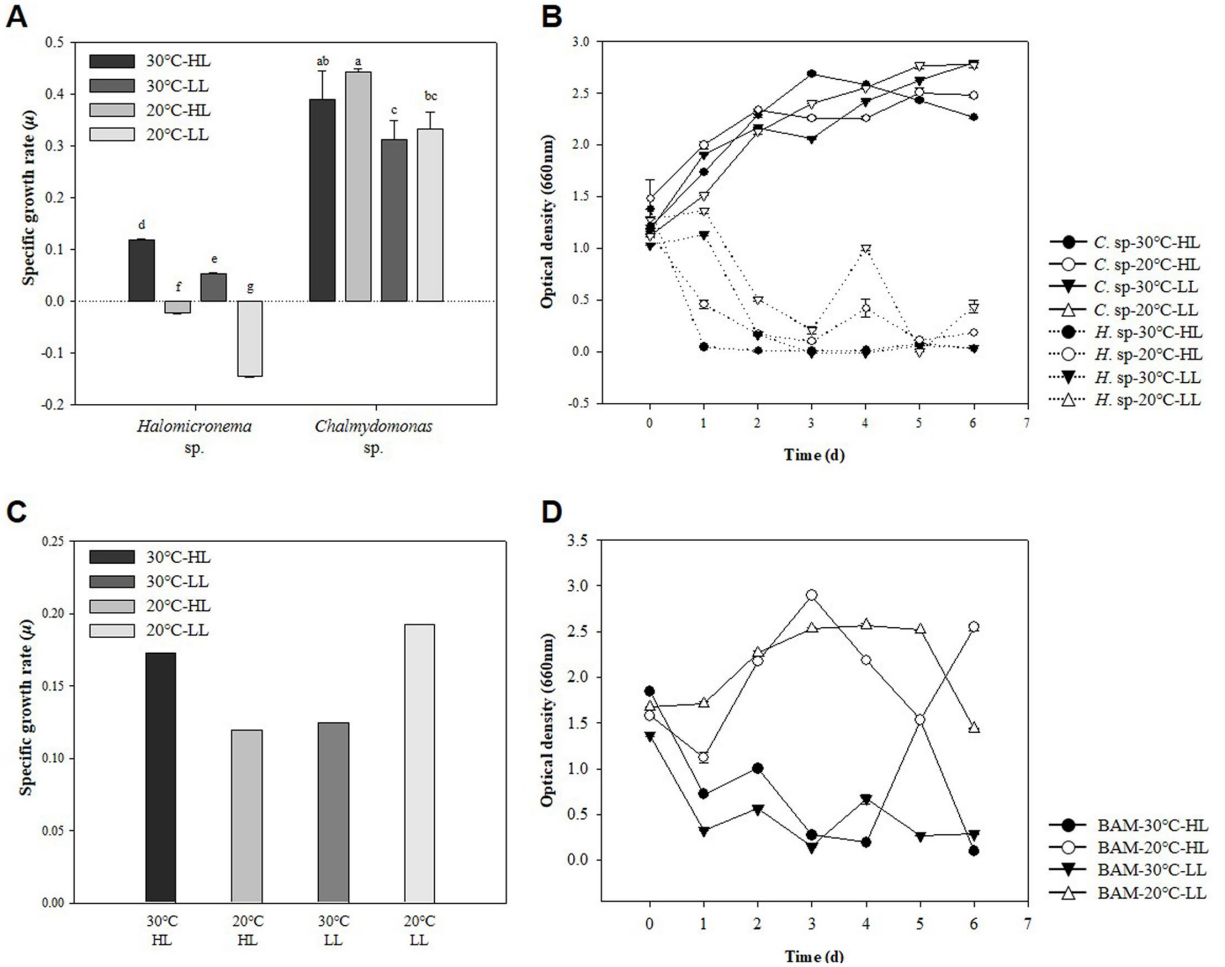
Strain characteristics of *Halomicronema* sp. and *Chlamydomonas* sp.: **(A)** DCW in pure culture. The above characters (a–g) are statistical comparisons between groups using ANOVA to determine significant differences (*p* < 0.05), **(B)** OD in pure culture, **(C)** DCW in mixed culture, **(D)** OD in mixed culture (Dash line, *Halomicronema* sp.; Straight line, *Chlamydomonas* sp.).

Representative methods for measuring the growth of microalgae include measuring the DCW and the absorbance of cells (Lu et al., 2017). Generally, these two measurement results show similar curves as microalgae grow, but characteristics such as cell attachment or cell size change may cause different results (Kim et al., 2014). Conversely, when cells grow and aggregate, OD is rarely used to measure growth because it cannot substitute for growth. In this study, specific growth rate (SGR) and OD were analyzed to compare the specific growth rates of two species at HL (120 μmol m^−2^ s^−1^) and LL (60 μmol m^−2^ s^−1^) for *Halomicronema* sp. and *Chlamydomonas* sp. under high temperature and low temperature conditions of 30°C and 20°C. Therefore, the maximum specific growth rate of biomass was 0.119 d^−1^ at HL −30°C in the case of *Halomicronema* sp., and 0.442 d^−1^ in the case of *Chlamydomonas* sp. at HL −20°C condition (Figure 2A). None of *Halomicronema* sp. grew at 20°C, but *Chlamydomonas* sp. at LL −30°C showed a growth rate of 70% compared to the optimal conditions. Generally, microalgae grow remarkably under abundant light conditions, and both species grow better under HL conditions than under LL conditions (Bonente et al., 2012). Additionally, the growth rate of *Chlamydomonas* sp. according to the temperature was higher at 20°C than at 30°C. *Halomicronema* sp. showed a slower growth about 6 times lower than that reported by Li et al. (2014) agrees that *Halomicronema hongdechloris* requires more than 150 h of doubling time.

Morphologically, *Halomicronema* sp. initially floated, but over time, it attached to the center of the bottom of the flask and grew into a complex net-like structure (Supplementary material 1). This was presented as an absorbance value of 0.5, which was more than 50% less than that on the first day after 24 h of culture in the absorbance measurement (Figure 2B). As the incubation period increased, cells attached to the bottom extended sideways to form a thin plate-shaped biofilm. As a result, most cells were not floating and attached after 48 h of incubation, showing a very low absorbance value of ≤0.1. This is consistent with previous findings indicating that *Halomicronema* sp. grows into mat form by grouping thin filaments into layered bundles (Zupo et al., 2019). Therefore, *Halomicronema* sp. do not grow in a suspension state but grow attached to the inner surface, indicating a low absorbance value.

Conversely, *Chlamydomonas* sp. did not decrease in absorbance with growth, and the absorbance value for general algae continued to increase with growth. Instead, a transparent viscous substance was produced in the culture medium, and after a week of incubation, the substances clumped together like a lump. Bafana (2013) stated that *Chlamydomonas reinhardtii* can produce large amounts of EPS, particularly to increase its content under various conditions. Therefore, the two types of adhesive and viscous substances that appeared during the culture process were highly likely to be EPS; therefore, the EPS contents of the two species were compared.

#### 3.2.2. Changes in EPS content in pure culture

Generally, the EPS production and secretion mechanisms of microalgae are caused by stressors, among which light intensity and temperature are affected (Babiak and Krzemińska, 2021). EPS is mostly composed of polysaccharides and proteins, which were measured according to the culture conditions. They were measured separately as s-EPS released to the outside and b-EPS attached to cells according to the measurement location of EPS (Kim et al., 2021). As a result of incubating *Chlamydomonas* sp. and *Halomicronema* sp. for 7 days under different light and temperature conditions, culturing *Chlamydomonas* sp. at LL −20°C increased by approximately 2.7 times, exhibiting the highest productivity of polysaccharide s-EPS (Table 1). At this time, the protein content hardly increased; therefore, in the case of *Chlamydomonas* sp., most EPS were composed of polysaccharides. Furthermore, *Chlamydomonas* sp. was found to be capable of producing a large amount of s-EPS. From the perspective of the EPS release mechanism, which Ramus (1972) reported, EPS bound outside the cell dissolves into a culture, the b-EPS content increases before the increase in s-EPS content, and the viscous material in the culture identified in the previous experiment was clumped up with the cell. In contrast, in the case of polysaccharide b-EPS, *Halomicronema* sp. showed the highest production 5.5 times under most conditions regardless of temperature, which was higher than the *Chlamydomonas* sp. b-EPS production rate of 4.3 times. In addition, unlike *Chlamydomonas* sp., which consists mostly of polysaccharides with a protein content of up to 21 mg L^−1^, *Halomicronema* sp. showed a protein content of up to 100 mg L^−1^, indicating the high productivity of b-EPS with high protein content. Given the finding that high b-EPS content provides functionality in connection with the aggregation and adhesion of microalgae, the fast attachment characteristics of cells in previous studies are thought to be related to b-EPS content (Lai et al., 2018). *Halomicronema* sp. was identified as a new strain; therefore, only a few genetic and phylogenetic studies have been conducted on it (Abed et al., 2002; Caroppo et al., 2012). In addition, studies have focused on extracellular polysaccharides among the extracellular substances of various cyanobacteria (Yang and Kong, 2013). However, despite their potential importance, studies on EPS in cyanobacteria have focused on analyzing the total protein content and amino acid composition, and few biochemical studies have focused on the protein component. The protein content of cyanobacteria is important for determining cell cohesiveness; therefore, further research is necessary.

**Table 1:** Production of soluble EPS and bound EPS of *Halomicronema* sp., *Chlamydomonas* sp. and BAM during 7 days of cultivation.

Polysaccharide	s-EPS (mg L^−1^)				b-EPS (mg L^−1^)			
	HL		LL		HL		LL	
	1 days	7 days	1 days	7 days	1 days	7 days	1 days	7 days
30°C-H	65.4 ± 6.2	47.05 ± 11.2	65.4 ± 6.2	187.9 ± 8.52	38.05 ± 0.91	206.1 ± 0.17	38.05 ± 0.91	210.6 ± 2.27
20°C-H	65.4 ± 6.2	82.00 ± 8.11	65.4 ± 6.2	97.84 ± 56.5	38.05 ± 0.91	191.7 ± 0.57	38.05 ± 0.91	210.6 ± 1.32
30°C-C	161.7 ± 0.29	171.1 ± 0.03	161.7 ± 0.29	330.6 ± 5.50	64.14 ± 0.16	156.1 ± 1.63	64.14 ± 0.16	280.2 ± 1.32
20°C-C	161.7 ± 0.29	175.1 ± 0.06	161.7 ± 0.29	436.7 ± 4.61	64.14 ± 0.16	146.6 ± 0.60	64.14 ± 0.16	190.8 ± 1.67
30°C-BAM	368.7 ± 0.66	282.3 ± 1.61	368.7 ± 0.66	295.8 ± 1.63	102.1 ± 0.67	1163 ± 13.24	102.1 ± 0.67	984.6 ± 4.00
20°C-BAM	368.7 ± 0.66	319.8 ± 0.29	368.7 ± 0.66	327.7 ± 3.69	102.1 ± 0.67	767.3 ± 7.01	102.1 ± 0.67	1199 ± 4.29
Protein	s-EPS (mg L^−1^)				b-EPS (mg L^−1^)			
	HL		LL		HL		LL	
	1 days	7 days	1 days	7 days	1 days	7 days	1 days	7 days
30°C-H	0.00 ± 0.00	12.29 ± 0.69	0.00 ± 0.00	10.50 ± 1.49	0.00 ± 0.00	72.63 ± 0.82	0.00 ± 0.00	100.2 ± 0.50
20°C-H	0.00 ± 0.00	2.37 ± 0.30	0.00 ± 0.00	7.82 ± 1.17	0.00 ± 0.00	75.39 ± 0.53	0.00 ± 0.00	85.30 ± 28.1
30°C-C	0.00 ± 0.00	3.90 ± 0.14	0.00 ± 0.00	5.71 ± 1.63	0.00 ± 0.00	2.99 ± 0.33	0.00 ± 0.00	4.72 ± 0.11
20°C-C	0.00 ± 0.00	0.00 ± 0.00	0.00 ± 0.00	0.00 ± 0.00	0.00 ± 0.00	0.00 ± 0.00	0.00 ± 0.00	18.80 ± 0.17
30°C-BAM	0.00 ± 0.00	9.03 ± 0.62	0.00 ± 0.00	4.24 ± 0.95	0.00 ± 0.00	44.38 ± 20.5	0.00 ± 0.00	49.55 ± 2.87
20°C-BAM	0.00 ± 0.00	27.88 ± 2.47	0.00 ± 0.00	19.39 ± 0.67	0.00 ± 0.00	134.3 ± 3.18	0.00 ± 0.00	76.28 ± 0.71

Generally, the EPS productivity of microalgae increases under stressful conditions, such as high light sources or temperatures (Babiak and Krzemińska, 2021), and Wang et al. (2019) reported that s-EPS production becomes active even in limited nutritional environments. In this study, *Chlamydomonas* sp. showed the highest biomass productivity and EPS productivity under LL −20°C conditions; therefore, EPS productivity is thought to have increased owing to the depletion of nutrients caused by rapid cell growth. In addition, the protein content in EPS is rarely measured and is below 3% of the polysaccharide content, indicating that the s-EPS produced by *Chlamydomonas* sp. is mainly composed of polysaccharides. The extracellular substances produced by *Chlamydomonas* sp. typically contains a higher proportion of polysaccharides than proteins (Li et al., 2019, 2021). Most *Chlamydomonas* genera have reported little protein content in EPS, which is consistent with the analysis results indicating that the protein content of *Chlamydomonas augustae* is within 4% of the polysaccharide content (Allard and Tazi, 1992).

### 3.3. Mixed culture of *Halomicronema* sp. and *Chlamydomonas* sp.

#### 3.3.1. Growth biomass and optical density

Previous experiments have resulted in cell-clumped EPS clumps owing to the high b-EPS of *Halomicronema* sp. and high s-EPS productivity of *Chlamydomonas* sp. This form was very similar to that first collected in the wild. Owing to a certain mechanism, we attempted to imitate naturally derived mats to elucidate the characteristics of algal mats that aggregate to form lumps in the wild. Using the EPS lump constituting *Chlamydomonas* sp., we attempted to determine the possibility of producing a new type of BAM grown by attaching *Halomicronema* sp. As a result of incubation, the highest biomass productivity was exhibited at 1.26 and 1.47 g L^−1^ under HL −30°C and LL −20°C conditions, respectively (Figure 2C). Absorbance at LL −20°C, with the highest biomass productivity, continuously increased to ≥1.44 after 24 h of culture, but tended to continuously decrease to 0.5 level after 24 h at HL −30°C (Figure 2D). These results indicated biomass productivity was highest at LL −20°C condition but did not show BAM formation using the agglomerated EPS mass of *Chlamydomonas* sp.

Conversely, at HL −30°C, the absorbance value was reduced and maintained similar to the previous culture of *Halomicronema* sp. alone, indicating that even when *Chlamydomonas* sp. was mixed, it aggregated in the form of a BAM. Although some small flocs are formed in mixed cultures of microalgae (Wrede et al., 2014), BAM, a new type of biofilm we induced, is a shape in which filamentous algae cover and grow over it using a mixed form of *Chlamydomonas* sp. and EPS as a support. In particular, after BAM formation, cell detachment from the culture medium was not confirmed, and low absorbance values were maintained. This is similar to the appearance observed when the filamentous body was covered with sludge in a recent photographic study (Milferstedt et al., 2017), where instead of sludge, the lump of *Chlamydomonas* sp. acted as a substrate. Considering that the firmly aggregated BAM was formed under the favorable HL −30°C conditions for the growth of *Halomicronema* sp. and showed lower absorbance, the growth of *Halomicronema* sp. was an important factor in the formation of the BAM of the two species.

#### 3.3.2. Changes in EPS content in mixed culture

Characteristic changes in EPS during mixed culture were confirmed to produce 10 times more polysaccharide b-EPS than in the initial stage when BAM was formed (Table 1). This yielded a production value of 1199 mg L^−1^, which is higher than that when culturing *Chlamydomonas* sp. Alone (436 mg L^−1^), which is the highest previously shown carbohydrate EPS production value. At this time, considering that the protein value of b-EPS ranges from 44 to 136 mg L^−1^, which is not higher than that of the existing culture of *Halomicronema* sp., but similar or lower, most of it is EPS produced by *Chlamydomonas* sp. The s-EPS concentration was higher in the single culture, but the b-EPS concentration was higher in the mixed culture; therefore, the mixed culture with *Halomicronema* sp. affected the release of EPS. Wang et al. (2019) explained that the production of b-EPS does not directly affect the weight of biomass; however, the stable protein content in b-EPS builds a crosslinked network to maintain the internal biological membrane structure, induces rapid biosynthesis of EPS polysaccharides, and enhances microbial adhesion. Arcila and Buitrón (2017) demonstrated that an increase in the protein/polysaccharide (PN/PS) ratio of EPS has a positive effect on the formation of flocs and granules in microalgae-bacteria aggregates. Extracellular proteins (PNs) are crucial for maintaining the cohesion and long-term stability of biofilms and play an essential role in preserving the structural integrity of the EPS matrix (Kawaguchi and Decho, 2000; Xiong and Liu, 2013). In addition, Lubarsky et al. (2010) reported that when proteins in EPS interact with polysaccharides, they can form a very robust unique structure by forming an elastic matrix that is similar to epoxy resin. In this study, high protein b-EPS produced by *Halomycronema* sp. and polysaccharide-EPS produced by *Chlamydomonas* sp. were found to have a solid structure.

### 3.4. Identification of EPS and BAM formation of *Chlamydomonas* sp. in mixed culture

#### 3.4.1. Comparison of algal cells and EPS through zeta potential and FT-IR

**Figure 3:**
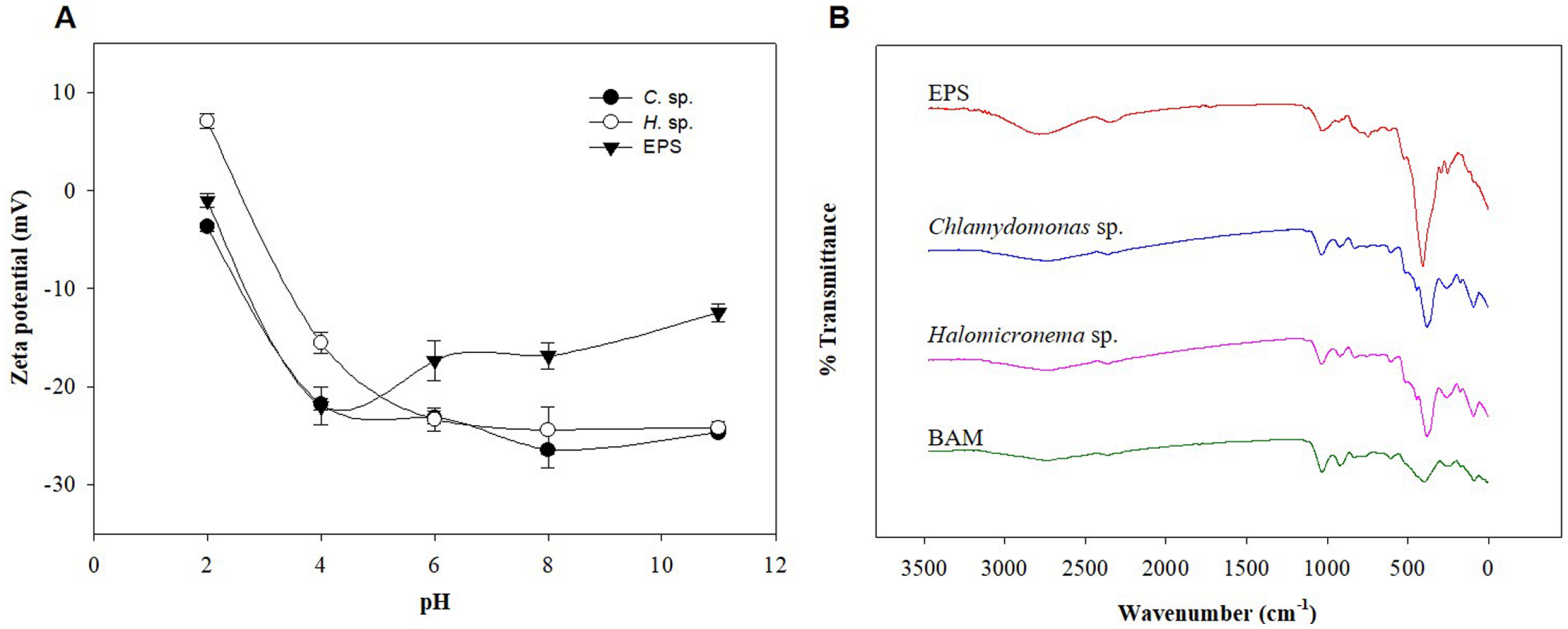
**(A)** Zeta potential of *Halomicronema* sp., *Chlamydomonas* sp. and EPS. **(B)** FTIR spectrum.

Flocculation of microalgae is typically caused by differences in pH and charge (Li et al., 2020). Microalgae are negatively charged photosynthetic organisms (Milledge and Heaven, 2013), and the EPS they produce and release are negatively charged (He et al., 2021). To understand the causes of BAM formation in the above study despite repulsive forces due to the same charge, *Halomicronema* sp., *Chlamydomonas* sp., and s-EPS produced by *Chlamydomonas* sp. were compared through zeta potential analysis. As a result, *Halomicronema* sp. showed a positive charge of 7.1 mV under pH 2 conditions, but a negative charge corresponding to −15.5 to −24.5 mV in the remaining pH intervals (Figure 3A). *Chlamydomonas* sp. showed negative charges at all pH intervals. In addition, s-EPS separated from *Chlamydomonas* sp. also showed negative charges at all pH intervals within the range of −1.0 to −22.0 mV. In the case of microalgal cells, the potential value rapidly decreased as the pH changed from 2 to 4, whereas in the case of the isolated s-EPS, the potential increased at pH 4 or higher. The zeta potential is used to indicate the degree of dispersion and agglomeration of particles; the higher the absolute value, the more optimized the dispersion state (Matho et al., 2019). Because the culture and growth of a general algal colony requires a pH of ≥7, the higher the pH, the lower the zeta potential value of EPS. This indicates that the low absolute value of EPS is favorable for aggregation. Aggregation by cationic inulin leads to electrostatic interactions between opposite charges, neutralizing negatively charged algae surfaces, reducing electrostatic repulsion between cells, destabilizing algae suspensions, and facilitating aggregation (Ummalyma et al., 2017). The EPS of microalgae has many functional groups and can remove various cationic substances such as heavy metals (Li and Yu, 2014). In addition, various cations such as Ca^2+^ and Mg^2+^ are present in the general culture medium (Stanier et al., 1971; Fábregas et al., 2000). Therefore, each functional group was compared using FT-IR to predict the possibility that the cationic component attached to the EPS and imparted anionic property was lower than that of the cell.

FT-IR was used to identify the structural bonding information of various functional groups and metal cations in EPS owing to the adsorption characteristics of EPS (Comte et al., 2006). Accordingly, the IR bands of BAM, s-EPS obtained from *Chlamydomonas* sp., and each microalgal cell were classified and compared according to existing literature (Comte et al., 2006). According to analysis results, the four samples showed consistent patterns (Figure 3B). The peaks at 1031–1077 cm^−1^ and 1605–1645 cm^−1^ are polysaccharide functional and protein (peptide bond) functional groups, respectively. Driver et al. (2015), defined protein-related C=O vibrations in FT-IR analysis of *Chlamydomonas reinhardtii* as amide I peaks at 1655 cm^−1^, and carbohydrates were observed below 1100 cm^−1^. However, the FT-IR analysis results showed that the peak in the 1000 cm^−1^ band differed in terms of quality, which is a polysaccharide structure of O-H stretching vibration, very deep in EPS, and conflicting results were shown in BAM. This appears to have led to a reduction in the strong polysaccharide structure in EPS during the formation of single cells into BAM. In particular, 1040 cm^−1^ was used as the diagnostic spectrum peak of hemicellulose (Hong et al., 2021), and the EPS component derived from *Chlamydomonas* sp. accounted for most sugar-related functional groups. These results show that polysaccharide-based EPS considerably contribute to BAM formation, and it is necessary to determine whether s-EPS directly helps attach to BAM formation. Nevertheless, additional studies were conducted based on the zeta potential and FT-IR analysis, as cations such as calcium supplied to the EPS functional group and medium reacted with the functional group.

#### 3.4.2. Adhesion of Ca^2+^ to algal cell surfaces

**Figure 4:**
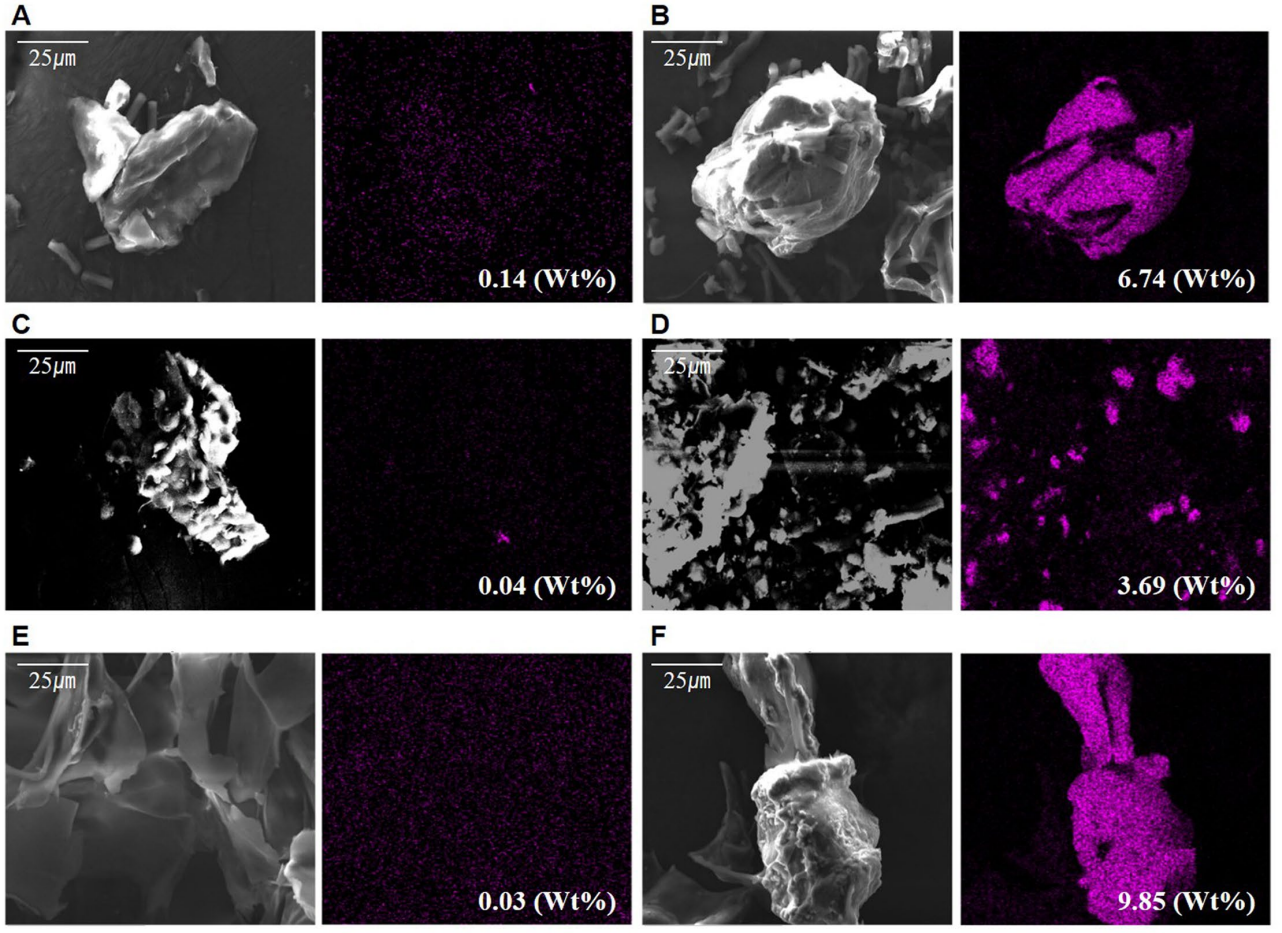
Scanned electron microscope observation photographs with differing calcium supply: **(A)** Calcium not supplied in *Halomicronema* sp., **(B)** Calcium supplied in *Halomicronema* sp., **(C)** Calcium not supplied in *Chlamydomonas* sp., **(D)** Calcium supplied in *Chlamydomonas* sp., **(E)** Calcium not supplied in EPS, **(F)** Calcium supplied in EPS.

The cation of the polymer is a prerequisite for biomass aggregation (Suresha and Badiger, 2019). Based on Babiak and Krzemińska (2021), the aggregation process varies depending on the presence of cations such as Ca^2+^ and Mg^2+^, which act as crosslinks with other polymers. The difference in adhesion between the two types of microalgae cells and the extracted s-EPS surface when adding cationic material was determined. The addition of a large amount of Ca^2+^ ions under the same conditions as the existing culture was compared to no addition. Following the confirmation of Ca^2+^ on each surface of the sample using EDS-SEM, a very low ratio of Ca^2+^ was found to be distributed in the control without Ca^2+^, and the experiment with Ca^2+^ ions was high on the outside of the cell wall (Figures 4A–F and Supplementary material 2). In particular, in the EPS sample, the maximum rate was 0.03% in the non-supply test zone, which was very low, but 9.85% in the supplied test zone, which was significantly higher than that of the experimental groups.

These results show that it can be attached to cells and EPS through a sufficient supply of cationic materials, thereby lowering the negative chargeability. This is particularly most common in the EPSs as they exhibit the highest adsorption level. Moreover, the charge repulsive force could be reduced through EPS, which is capable of attaching a large number of Ca^2+^ ions to the surface. In addition, *Halomicronema* sp., which produces more b-EPS on the cell surface than s-EPS, which is relatively weak in adhesion, plays an important role in aggregation.

Babiak and Krzemińska (2021) reported that the adsorption of Ca^2+^ and Mg^2+^ to EPS can partially neutralize the electronegativity of EPS, suggesting that microalgae-derived EPS, which holds a large amount of supplied positive charges, plays an additional role in maintaining surface bonding between negatively charged cells.

#### 3.4.3. Relationship between the mobility of *Halomicronema* sp. and BAM maintenance in mixed cultivation

Filamentous microalgae are mobile and can thus attach to the medium (Kurjahn et al., 2022), allowing them to select sites with optimal light and carbon (Milferstedt et al., 2017). Previous studies confirmed that *Halomicronema* sp. can form BAM using *Chlamydomonas* sp., which produces a large amount of EPS. However, the biofilm formed by *Halomicronema* sp. alone yielded a low absorbance value in BAM compared to the case where the cell leaves the mat when nutrients are depleted; the parts that maintain BAM for a long time need to be studied further.

**Figure 5:**
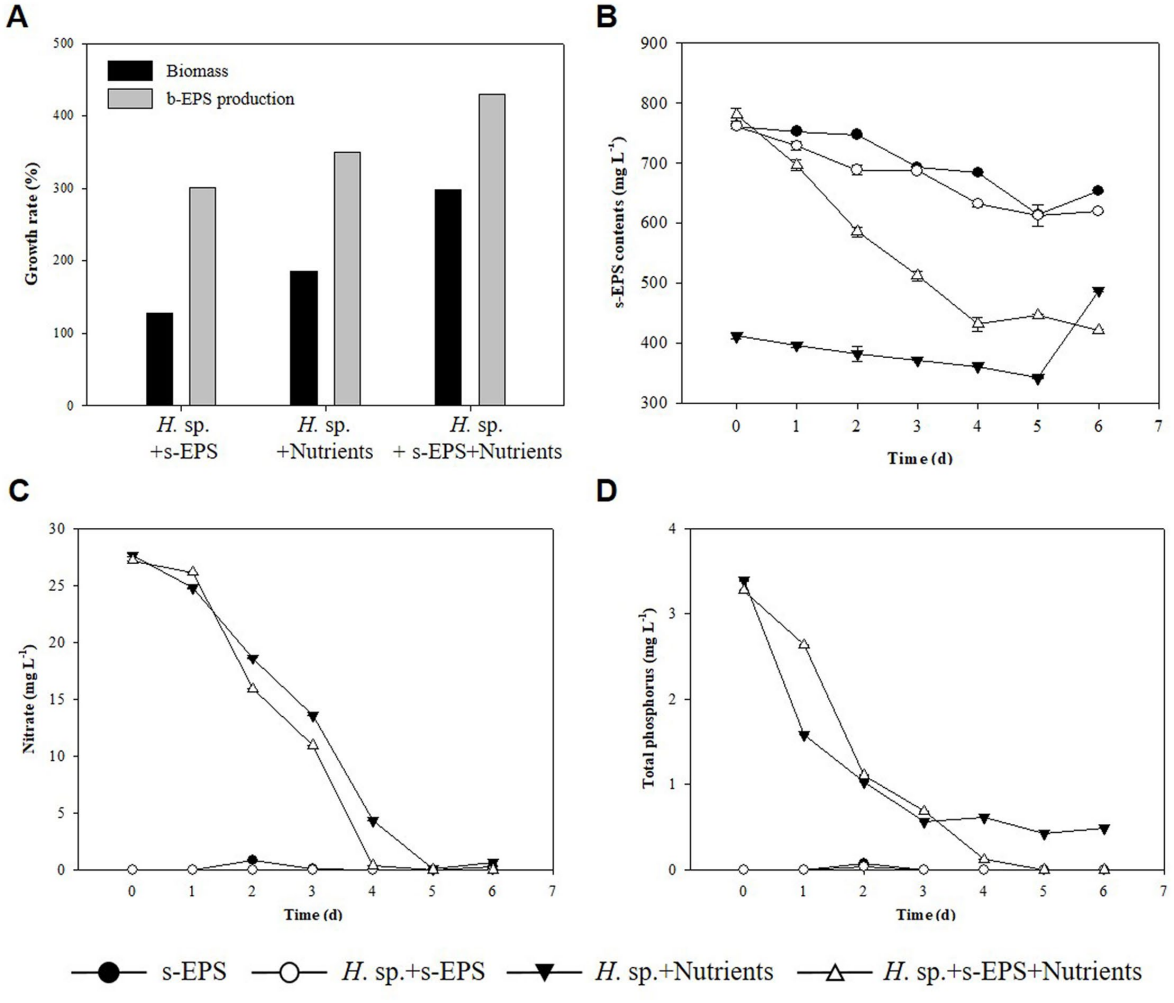
**(A)** Biomass growth rate (black) and b-EPS production content (gray). **(B)** s-EPS production contents. **(C)** TN. **(D)** TP.

In Table 1, we estimated that the decrease in s-EPS occurred during the culture mixing of the two strains, whereas that in s-EPS was affected by the growth of *Halomicronema* sp. To confirm this, only the s-EPS produced by *Chlamydomonas* sp. was extracted and added to the culture of *Halomicronema* sp. to compare the experimental conditions without adding s-EPS or upon adding a medium containing nitrogen and phosphorus. As a result of the experiment, the culture using only s-EPS without a medium grew by 127% for a week, exhibiting the lowest growth rate (Figure 5A). The growth of the experimental group that supplied only the medium without s-EPS increased by 185%, and the experimental group that simultaneously supplied the s-EPS and medium reported the highest growth (298%). To determine the consumption of s-EPS during *Halomicronema* sp. culturing, s-EPS was supplied and the decrease in the amount produced by *Halomicronema* sp. was confirmed (Figure 5B). However, the amount of s-EPS produced was estimated based on the experimental group inoculated with nutrients, without any additional s-EPS. The value of s-EPS in the culture decreased by 15% in both the experimental group without *Halomicronema* sp. and the experimental group with only *Halomicronema* sp., but decreased by more than 45% over 4 days the experimental group with *Halomicronema* sp. and s-EPS. The depletion of nitrogen and phosphorus nutrients was confirmed after 4 days of culture (Figures 5C,D). Because there was little reduction in s-EPS, the decrease in s-EPS was highly correlated with the decrease in nutrients. Comparison of biomass and b-EPS growth rates following nutrient supply and s-EPS based on biomass growth rate and b-EPS growth rate under conditions supplied with only nutrients showed that b-EPS grew by 123%, and biomass by 161%. Therefore, as mentioned above, the b-EPS protein of *Halomicronema* sp. and the polysaccharide s-EPS of *Chlamydomonas* sp. were combined to confirm direct cell biomass growth, not the increase in biomass. Certain microalgae can utilize EPS as a nutrient (De Brouwer and Stal, 2002), and organic substances such as glucose are directly utilized to increase growth under other and mixed nutritional conditions (Kim and Hur, 2013). Therefore, although s-EPS does not replace the entire medium component, when supplied additionally with a medium containing nitrogen and phosphorus, it can increase the productivity of *Halomicronema* sp. in the form of organic matter supply. This suggests that *Halomicronema* sp. can be maintained for a long time without leaving the BAM because *Chlamydomonas* sp. continuously supplements s-EPS in the BAM formed by *Halomicronema* sp. and *Chlamydomonas* sp. Finally, the production of protein-type b-EPS with the growth of *Halomicronema* sp. plays an important role in the formation of solid clusters. *Halomicronema* sp. growth increases with the supply of s-EPS produced by the relatively fast growing *Chlamydomonas* sp. b-EPS and s-EPS combine to form a solid structure (Lubarsky et al., 2010), and s-EPS is not released to the outside and remains in the cluster. The remaining EPS is combined with the surrounding calcium ions to reduce electrostatic repulsion (Ummalyma et al., 2016), and we believe that the stability and growth of the BAM cluster are maintained through this effect.

### 3.5. Assessment of BAM buoyancy

A special phenomenon during the cultivation of BAM, comprising a mixture of two species at various temperatures, is the phenomenon where BAM has buoyancy and rises to the surface. Generally, algae are single-celled, suspended, or noncellular, and filamentous algae grow in a form attached to a specific medium (Zupo et al., 2019). Therefore, most microalgae were evenly distributed in the culture medium or attached to the bottom of the culture flask and grew widely. In this study, BAM, comprising a mixture of the two microalgae, was observed to float and grow, with numerous bubbles generated during the photosynthesis of microalgae between them.

**Figure 6:**
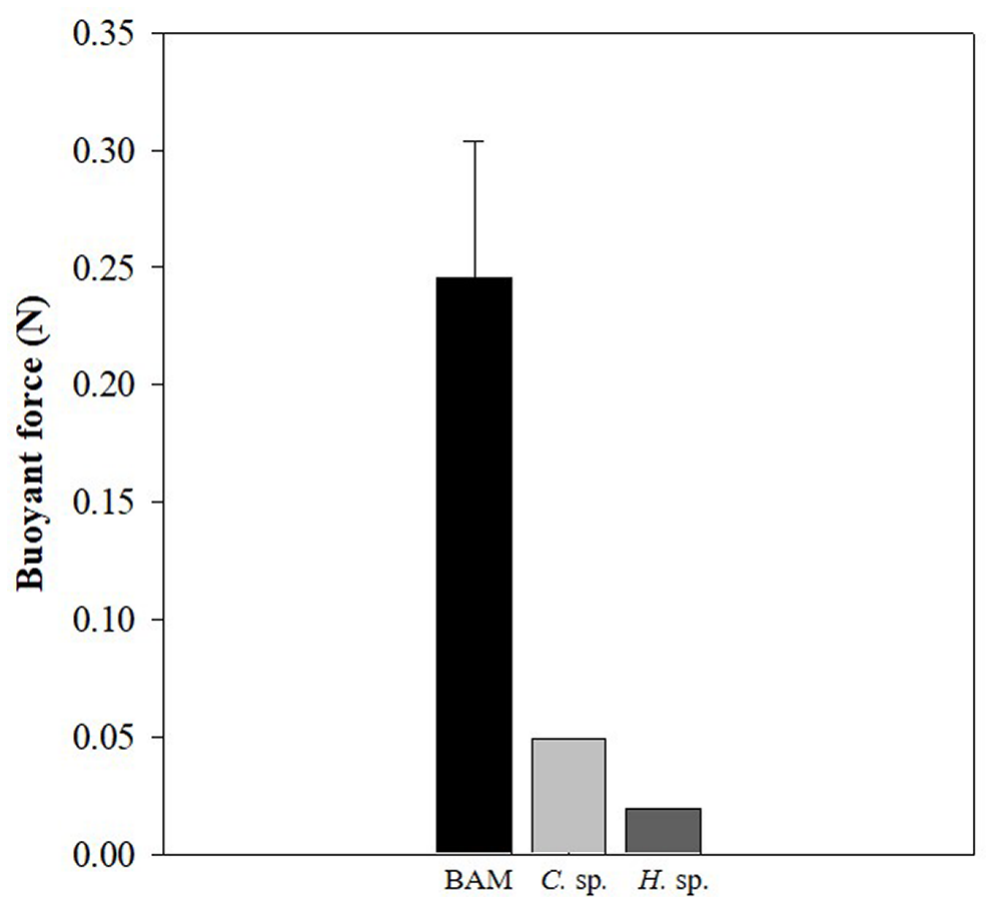
Difference in buoyancy between the BAM, *Chlamydomonas* sp., and *Halomicronema* sp.

The buoyancy of BAM and individual species was calculated and BAM was found to have the highest positive buoyancy at 0.25 ± 0.06 N, while those of *Chlamydomonas* sp. and *Halomicronema* sp. were 0.05 and 0.02 N, respectively (Figure 6). This indicates that the buoyancy increased substantially owing to the mixing of the two species.

**Figure 7:**
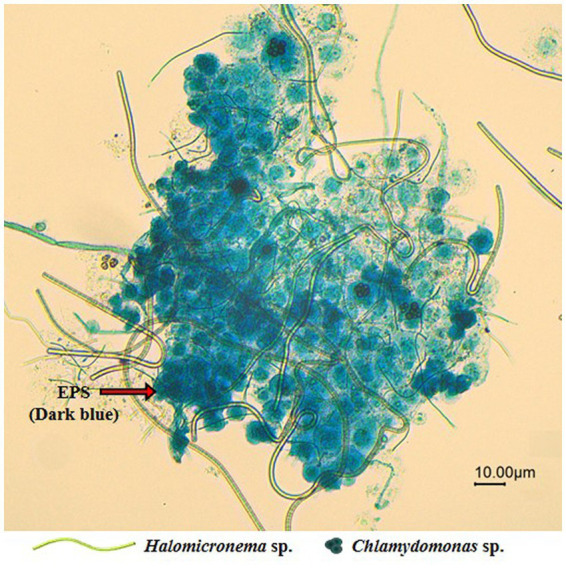
Microscope image (20X) of the EPS produced around the aggregated BAM.

Following the dyeing of EPS to facilitate observations of specific BAM shapes, we found that *Halomicronema* sp. are elongated and filamentous in shape and serve as the EPS matrix foundation. In contrast, *Chlamydomonas* sp. are circular algae that fill the spaces within the EPS matrix (Figure 7). In addition, the EPSs formed a wide matrix with high cohesion to cover the cells. Negative zeta potential with high absolute values prevents the stable dispersion of algal cells in the surrounding medium and their adherence to the bubble surface (Hao et al., 2017). However, EPS formed in large quantities around microalgal cells grant the culture environment zeta potential with low absolute values, leading to active aggregation and bubble adhesion. These features were not observed in individual species, but appear to be an important influencing factor for the high buoyancy of BAMs.

In addition, the use of s-EPS in the growth of *Halomicronema* sp., identified in our previous experimental results, appears to be one of the reasons underlying the formation of additional spaces through the consumption of carbohydrates that make up the matrix. At the same time, in the case of *Chlamydomonas* sp., EPS production was highest under low-light conditions, and when BAM was formed, *Chlamydomonas* sp. was covered by *Halomicronema* sp. and EPS, which is believed to be the cause of accelerated EPS production. Therefore, s-EPS, which has increased productivity, can be inferred to be a factor that can reinforce low growth potential, a process that circulates to highly cohesive b-EPS production.

**Figure 8:**
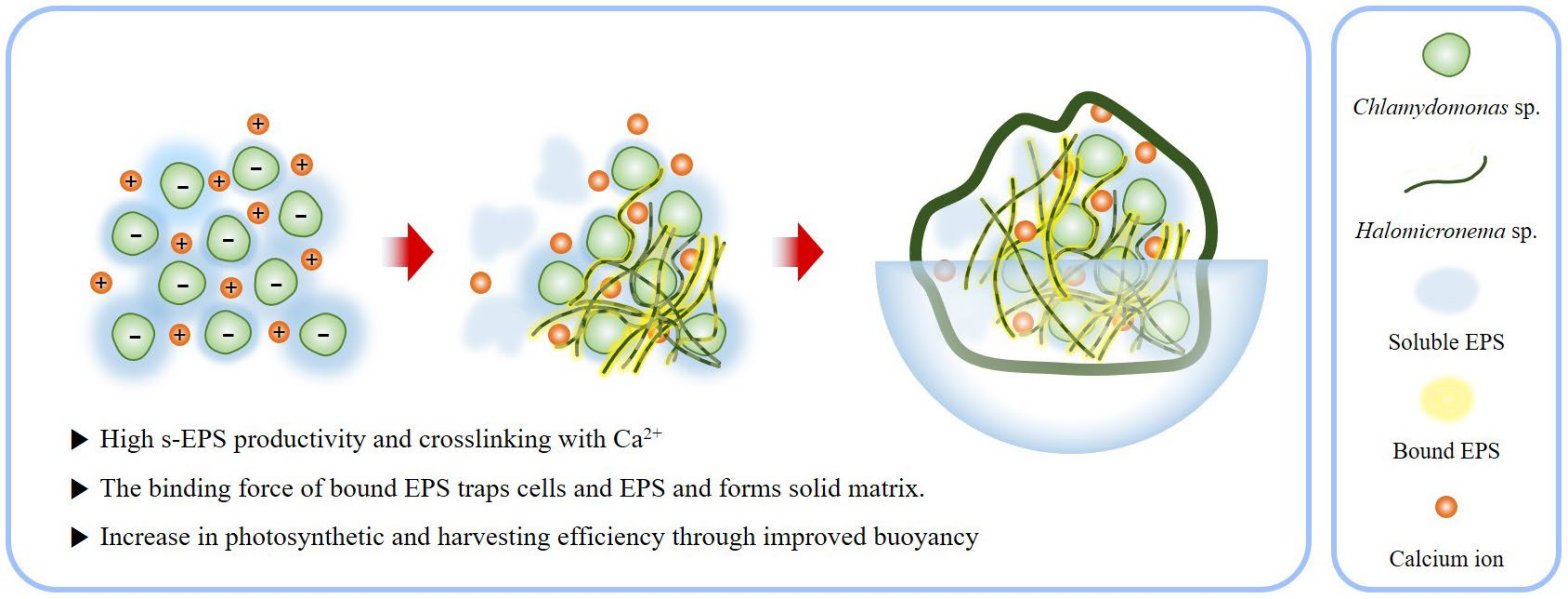
Schematic of process by which the EPS produced by *Chlamydomonas* sp. and *Halomicronema* sp. are mixed to form BAM.

As a result, BAM constituting the EPS matrix has several ecological advantages in buoyancy. By exposing the top of the colony to the surface, an active photosynthetic state can be maintained without any additional support or treatment (Figure 8). This provides an opportunity for the BAM to access a large amount of carbon dioxide by contacting the atmosphere. Furthermore, while the existing biofilm is harvested by precipitating algae, the characteristic of floating on the water surface makes harvesting much easier. Because of these advantages, BAMs could be directly applicable to contaminated areas, because the robust structures generated through the EPS interaction of *Chlamydomonas* sp. and *Halomicronema* sp. are unlikely to negatively affect the surrounding environment due to dispersal and diffusion. Thus, BAM shows the possibility of simultaneously purifying water quality and producing effective biomass.

## 4. Conclusion

Microbial mats collected from nature can form on their own without any artificial treatment, making them a valuable addition to biomass production technology. *Halomicronema* sp. and *Chlamydomonas* sp. were used to determine the formation mechanism of a natural microalgal mat, and the role of EPS. *Halomicronema* sp. was grown to be a biofilm mat by attaching EPS produced by *Chlamydomonas* sp. and cell mass as a medium. Furthermore, the low growth potential of *Halomicronema* sp. was improved using the s-EPS of polysaccharides produced by *Chlamydomonas* sp. A large amount of EPS combined with Ca^2+^ plays an important role in the formation and buoyancy of microalgal biofilm by controlling the surface charge of microalgae, and thereby maintaining a mat shape with enhanced aggregation for a long time and without the use of additional equipment or chemical treatment. The study proposes a new cultivation method that can produce biomass inexpensively and with low harvesting cost thus providing reference for the development of a sustainable and cost-effective method for the formation of a microalgal mat. However, our study was conducted at a laboratory scale and was not affected by changes in the external environment; therefore, it is important to validate our findings in a natural environment in future studies to better our understanding of additional factors, such as temperature fluctuations, exposure to sunlight, symbiotic relationships, and the potential for invasion by other organisms. In addition, the role of bacteria, which we excluded from this study, must be fully addressed to achieve full biomimicry.

## Data availability statement

The raw data supporting the conclusions of this article will be made available by the authors, without undue reservation.

## Author contributions

HEL designed the study, carried out the experiments, and wrote the manuscript. JHL contributed to data analysis and designed the figures. SMP contributed to data analysis. DGK supervised the experiments and revised the manuscript. All authors in this study reviewed the manuscript and commented on the manuscript.

## Funding

This work was supported by the Korea Environmental Industry & Technology Institute (KEITI) through the Ecological Imitation-based Environmental Pollution Management Technology Development Project funded by the Korea Ministry of Environment (MOE) (2019002790009).

## Conflict of interest

The authors declare that the research was conducted in the absence of any commercial or financial relationships that could be construed as a potential conflict of interest.

## Publisher’s note

All claims expressed in this article are solely those of the authors and do not necessarily represent those of their affiliated organizations, or those of the publisher, the editors and the reviewers. Any product that may be evaluated in this article, or claim that may be made by its manufacturer, is not guaranteed or endorsed by the publisher.
