# Supplementary material for: Symbiotic relationship between filamentous algae (Halomicronema sp.) and extracellular polymeric substance-producing algae (Chlamydomonas sp.) through biomimetic simulation of natural algal mats
Source: Front Microbiol. 2023 May 24;14:1176069. doi: 10.3389/fmicb.2023.1176069 (PMC10244577; doi:10.3389/fmicb.2023.1176069)
Supplement: Supplementary file 1 [file Data_Sheet_1.docx]

**Supplementary 1.** **The culture of microalgae used in this study over time.**


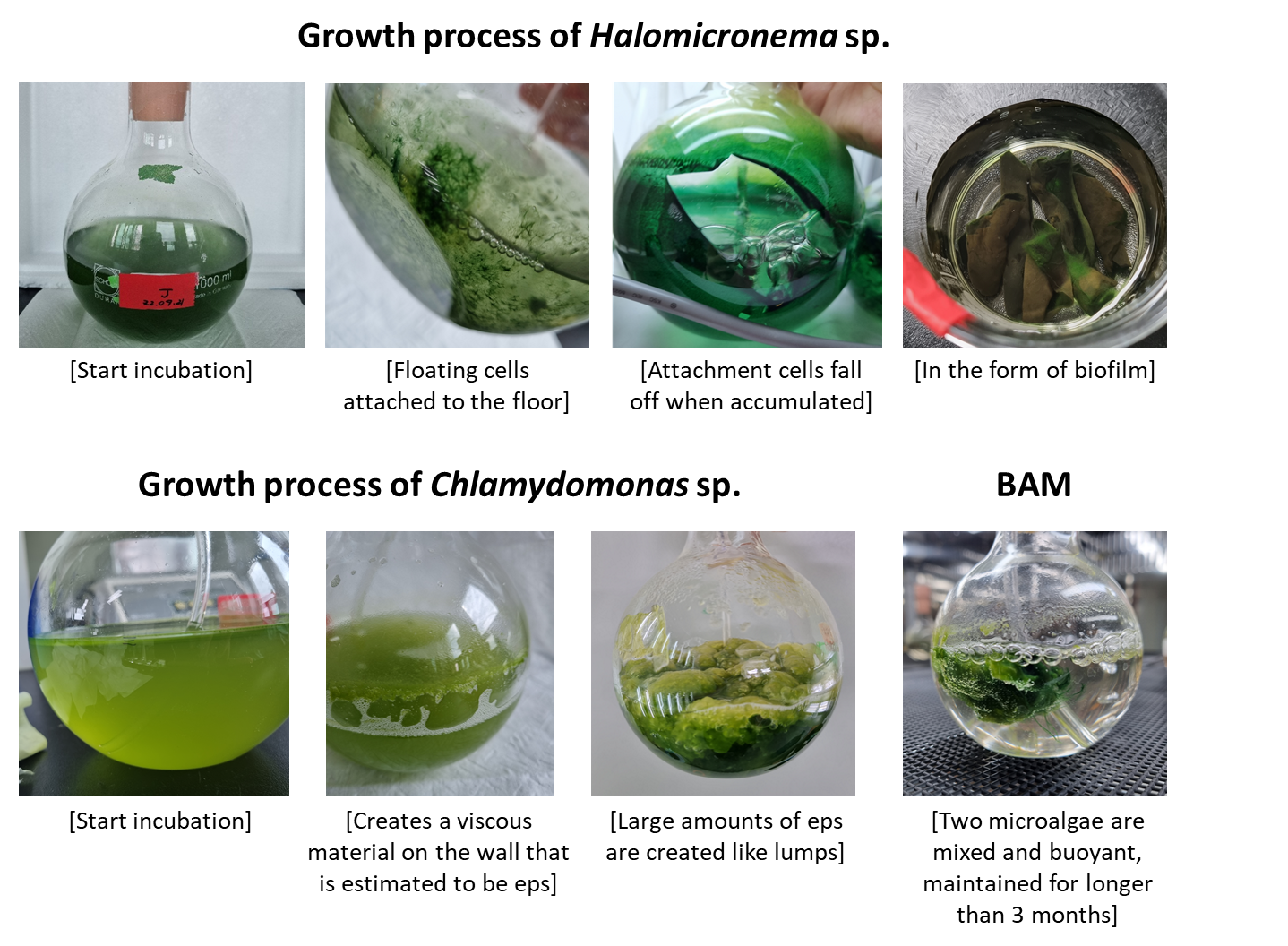


| **Supplementary 2. Calcium adhesion ratio results from observation and measurement of EDS-SEM.**  Figure 4A   \| Element \| Line Type \| Apparent Concentration \| k Ratio \| Wt% \| Wt% Sigma \| Atomic % \| Standard Label \| Factory Standard \| Standard Calibration Date \| \| --- \| --- \| --- \| --- \| --- \| --- \| --- \| --- \| --- \| --- \| \| C \| K series \| 24.21 \| 0.24208 \| 74.59 \| 0.18 \| 79.94 \| C Vit \| Yes \|  \| \| O \| K series \| 7.67 \| 0.02582 \| 24.46 \| 0.18 \| 19.68 \| SiO2 \| Yes \|  \| \| Na \| K series \| 0.03 \| 0.00011 \| 0.03 \| 0.02 \| 0.02 \| Albite \| Yes \|  \| \| Mg \| K series \| 0.08 \| 0.00056 \| 0.12 \| 0.01 \| 0.06 \| MgO \| Yes \|  \| \| Si \| K series \| 0.01 \| 0.00012 \| 0.02 \| 0.01 \| 0.01 \| SiO2 \| Yes \|  \| \| P \| K series \| 0.25 \| 0.00137 \| 0.22 \| 0.02 \| 0.09 \| GaP \| Yes \|  \| \| S \| K series \| 0.14 \| 0.00125 \| 0.20 \| 0.02 \| 0.08 \| FeS2 \| Yes \|  \| \| Cl \| K series \| 0.01 \| 0.00007 \| 0.01 \| 0.01 \| 0.00 \| NaCl \| Yes \|  \| \| K \| K series \| 0.16 \| 0.00131 \| 0.21 \| 0.02 \| 0.07 \| KBr \| Yes \|  \| \| Ca \| K series \| 0.11 \| 0.00094 \| 0.14 \| 0.02 \| 0.05 \| Wollastonite \| Yes \|  \| \| Total: \|  \|  \|  \| 100.00 \|  \| 100.00 \|  \|  \|  \|   Figure 4B   \| Element \| Line Type \| Apparent Concentration \| k Ratio \| Wt% \| Wt% Sigma \| Atomic % \| Standard Label \| Factory Standard \| Standard Calibration Date \| \| --- \| --- \| --- \| --- \| --- \| --- \| --- \| --- \| --- \| --- \| \| C \| K series \| 19.32 \| 0.19318 \| 59.86 \| 0.20 \| 69.17 \| C Vit \| Yes \|  \| \| O \| K series \| 12.15 \| 0.04090 \| 32.27 \| 0.18 \| 27.99 \| SiO2 \| Yes \|  \| \| Na \| K series \| 0.04 \| 0.00016 \| 0.04 \| 0.02 \| 0.03 \| Albite \| Yes \|  \| \| Mg \| K series \| 0.11 \| 0.00072 \| 0.14 \| 0.01 \| 0.08 \| MgO \| Yes \|  \| \| Si \| K series \| 0.03 \| 0.00024 \| 0.03 \| 0.01 \| 0.02 \| SiO2 \| Yes \|  \| \| P \| K series \| 0.34 \| 0.00188 \| 0.26 \| 0.02 \| 0.12 \| GaP \| Yes \|  \| \| S \| K series \| 0.22 \| 0.00194 \| 0.27 \| 0.02 \| 0.12 \| FeS2 \| Yes \|  \| \| Cl \| K series \| 0.16 \| 0.00139 \| 0.19 \| 0.02 \| 0.08 \| NaCl \| Yes \|  \| \| K \| K series \| 0.17 \| 0.00143 \| 0.19 \| 0.02 \| 0.07 \| KBr \| Yes \|  \| \| Ca \| K series \| 5.71 \| 0.05100 \| 6.74 \| 0.05 \| 2.33 \| Wollastonite \| Yes \|  \| \| Total: \|  \|  \|  \| 100.00 \|  \| 100.00 \|  \|  \|  \| |
| --- | --- | --- | --- | --- | --- | --- | --- | --- | --- | --- | --- | --- | --- | --- | --- | --- | --- | --- | --- | --- | --- | --- | --- | --- | --- | --- | --- | --- | --- | --- | --- | --- | --- | --- | --- | --- | --- | --- | --- | --- | --- | --- | --- | --- | --- | --- | --- | --- | --- | --- | --- | --- | --- | --- | --- | --- | --- | --- | --- | --- | --- | --- | --- | --- | --- | --- | --- | --- | --- | --- | --- | --- | --- | --- | --- | --- | --- | --- | --- | --- | --- | --- | --- | --- | --- | --- | --- | --- | --- | --- | --- | --- | --- | --- | --- | --- | --- | --- | --- | --- | --- | --- | --- | --- | --- | --- | --- | --- | --- | --- | --- | --- | --- | --- | --- | --- | --- | --- | --- | --- | --- | --- | --- | --- | --- | --- | --- | --- | --- | --- | --- | --- | --- | --- | --- | --- | --- | --- | --- | --- | --- | --- | --- | --- | --- | --- | --- | --- | --- | --- | --- | --- | --- | --- | --- | --- | --- | --- | --- | --- | --- | --- | --- | --- | --- | --- | --- | --- | --- | --- | --- | --- | --- | --- | --- | --- | --- | --- | --- | --- | --- | --- | --- | --- | --- | --- | --- | --- | --- | --- | --- | --- | --- | --- | --- | --- | --- | --- | --- | --- | --- | --- | --- | --- | --- | --- | --- | --- | --- | --- | --- | --- | --- | --- | --- | --- | --- | --- | --- | --- | --- | --- | --- | --- | --- | --- | --- | --- | --- | --- | --- | --- | --- | --- | --- | --- | --- | --- | --- | --- |

| Figure 4C   \| Element \| Line Type \| Apparent Concentration \| k Ratio \| Wt% \| Wt% Sigma \| Standard Label \| Factory Standard \| Standard Calibration Date \| \| --- \| --- \| --- \| --- \| --- \| --- \| --- \| --- \| --- \| \| C \| K series \| 37.23 \| 0.37230 \| 77.69 \| 0.13 \| C Vit \| Yes \|  \| \| O \| K series \| 9.31 \| 0.03132 \| 22.01 \| 0.13 \| SiO2 \| Yes \|  \| \| Mg \| K series \| 0.12 \| 0.00076 \| 0.12 \| 0.01 \| MgO \| Yes \|  \| \| P \| K series \| 0.23 \| 0.00128 \| 0.15 \| 0.01 \| GaP \| Yes \|  \| \| Ca \| K series \| 0.04 \| 0.00038 \| 0.04 \| 0.01 \| Wollastonite \| Yes \|  \| \| Total: \|  \|  \|  \| 100.00 \|  \|  \|  \|  \|   Figure 4D   \| Element \| Line Type \| Apparent Concentration \| k Ratio \| Wt% \| Wt% Sigma \| Standard Label \| Factory Standard \| Standard Calibration Date \| \| --- \| --- \| --- \| --- \| --- \| --- \| --- \| --- \| --- \| \| C \| K series \| 12.88 \| 0.12881 \| 55.56 \| 0.49 \| C Vit \| Yes \|  \| \| N \| K series \| 2.60 \| 0.00463 \| 8.86 \| 0.72 \| BN \| Yes \|  \| \| O \| K series \| 8.34 \| 0.02808 \| 28.16 \| 0.30 \| SiO2 \| Yes \|  \| \| Mg \| K series \| 0.25 \| 0.00167 \| 0.38 \| 0.02 \| MgO \| Yes \|  \| \| P \| K series \| 0.67 \| 0.00374 \| 0.61 \| 0.02 \| GaP \| Yes \|  \| \| S \| K series \| 0.42 \| 0.00361 \| 0.60 \| 0.02 \| FeS2 \| Yes \|  \| \| Cl \| K series \| 0.89 \| 0.00778 \| 1.29 \| 0.03 \| NaCl \| Yes \|  \| \| K \| K series \| 0.62 \| 0.00529 \| 0.85 \| 0.03 \| KBr \| Yes \|  \| \| Ca \| K series \| 2.64 \| 0.02361 \| 3.69 \| 0.05 \| Wollastonite \| Yes \|  \| \| Total: \|  \|  \|  \| 100.00 \|  \|  \|  \|  \|   Figure 4E   \| Element \| Line Type \| Apparent Concentration \| k Ratio \| Wt% \| Wt% Sigma \| Atomic % \| Standard Label \| Factory Standard \| Standard Calibration Date \| \| --- \| --- \| --- \| --- \| --- \| --- \| --- \| --- \| --- \| --- \| \| C \| K series \| 10.12 \| 0.10125 \| 50.65 \| 0.32 \| 60.16 \| C Vit \| Yes \|  \| \| O \| K series \| 16.44 \| 0.05531 \| 41.48 \| 0.29 \| 36.99 \| SiO2 \| Yes \|  \| \| Mg \| K series \| 0.21 \| 0.00138 \| 0.31 \| 0.02 \| 0.18 \| MgO \| Yes \|  \| \| S \| K series \| 0.29 \| 0.00249 \| 0.40 \| 0.02 \| 0.18 \| FeS2 \| Yes \|  \| \| Cl \| K series \| 1.94 \| 0.01695 \| 2.73 \| 0.04 \| 1.10 \| NaCl \| Yes \|  \| \| K \| K series \| 0.81 \| 0.00690 \| 1.08 \| 0.03 \| 0.39 \| KBr \| Yes \|  \| \| Ca \| K series \| 1.77 \| 0.01583 \| 2.40 \| 0.05 \| 0.85 \| Wollastonite \| Yes \|  \| \| Zr \| L series \| 0.56 \| 0.00560 \| 0.94 \| 0.15 \| 0.15 \| Zr \| Yes \|  \| \| Total: \|  \|  \|  \| 100.00 \|  \| 100.00 \|  \|  \|  \|   Figure 4F   \| Element \| Line Type \| Apparent Concentration \| k Ratio \| Wt% \| Wt% Sigma \| Atomic % \| Standard Label \| Factory Standard \| Standard Calibration Date \| \| --- \| --- \| --- \| --- \| --- \| --- \| --- \| --- \| --- \| --- \| \| C \| K series \| 17.70 \| 0.17703 \| 59.57 \| 0.20 \| 70.13 \| C Vit \| Yes \|  \| \| O \| K series \| 9.66 \| 0.03252 \| 29.18 \| 0.19 \| 25.79 \| SiO2 \| Yes \|  \| \| Na \| K series \| 0.01 \| 0.00005 \| 0.01 \| 0.02 \| 0.01 \| Albite \| Yes \|  \| \| Mg \| K series \| 0.12 \| 0.00080 \| 0.16 \| 0.01 \| 0.10 \| MgO \| Yes \|  \| \| Si \| K series \| 0.06 \| 0.00044 \| 0.07 \| 0.01 \| 0.03 \| SiO2 \| Yes \|  \| \| P \| K series \| 0.18 \| 0.00100 \| 0.15 \| 0.02 \| 0.07 \| GaP \| Yes \|  \| \| S \| K series \| 0.18 \| 0.00152 \| 0.23 \| 0.02 \| 0.10 \| FeS2 \| Yes \|  \| \| Cl \| K series \| 0.45 \| 0.00391 \| 0.58 \| 0.02 \| 0.23 \| NaCl \| Yes \|  \| \| K \| K series \| 0.18 \| 0.00155 \| 0.22 \| 0.02 \| 0.08 \| KBr \| Yes \|  \| \| Ca \| K series \| 7.92 \| 0.07076 \| 9.85 \| 0.07 \| 3.48 \| Wollastonite \| Yes \|  \| \| Total: \|  \|  \|  \| 100.00 \|  \| 100.00 \|  \|  \|  \| |
| --- | --- | --- | --- | --- | --- | --- | --- | --- | --- | --- | --- | --- | --- | --- | --- | --- | --- | --- | --- | --- | --- | --- | --- | --- | --- | --- | --- | --- | --- | --- | --- | --- | --- | --- | --- | --- | --- | --- | --- | --- | --- | --- | --- | --- | --- | --- | --- | --- | --- | --- | --- | --- | --- | --- | --- | --- | --- | --- | --- | --- | --- | --- | --- | --- | --- | --- | --- | --- | --- | --- | --- | --- | --- | --- | --- | --- | --- | --- | --- | --- | --- | --- | --- | --- | --- | --- | --- | --- | --- | --- | --- | --- | --- | --- | --- | --- | --- | --- | --- | --- | --- | --- | --- | --- | --- | --- | --- | --- | --- | --- | --- | --- | --- | --- | --- | --- | --- | --- | --- | --- | --- | --- | --- | --- | --- | --- | --- | --- | --- | --- | --- | --- | --- | --- | --- | --- | --- | --- | --- | --- | --- | --- | --- | --- | --- | --- | --- | --- | --- | --- | --- | --- | --- | --- | --- | --- | --- | --- | --- | --- | --- | --- | --- | --- | --- | --- | --- | --- | --- | --- | --- | --- | --- | --- | --- | --- | --- | --- | --- | --- | --- | --- | --- | --- | --- | --- | --- | --- | --- | --- | --- | --- | --- | --- | --- | --- | --- | --- | --- | --- | --- | --- | --- | --- | --- | --- | --- | --- | --- | --- | --- | --- | --- | --- | --- | --- | --- | --- | --- | --- | --- | --- | --- | --- | --- | --- | --- | --- | --- | --- | --- | --- | --- | --- | --- | --- | --- | --- | --- | --- | --- | --- | --- | --- | --- | --- | --- | --- | --- | --- | --- | --- | --- | --- | --- | --- | --- | --- | --- | --- | --- | --- | --- | --- | --- | --- | --- | --- | --- | --- | --- | --- | --- | --- | --- | --- | --- | --- | --- | --- | --- | --- | --- | --- | --- | --- | --- | --- | --- | --- | --- | --- | --- | --- | --- | --- | --- | --- | --- | --- | --- | --- | --- | --- | --- | --- | --- | --- | --- | --- | --- | --- | --- | --- | --- | --- | --- | --- | --- | --- | --- | --- | --- | --- | --- | --- | --- | --- | --- | --- | --- | --- | --- | --- | --- | --- | --- | --- | --- | --- | --- | --- | --- | --- | --- | --- | --- | --- | --- | --- | --- | --- | --- | --- | --- | --- | --- | --- | --- | --- | --- | --- | --- | --- | --- | --- | --- | --- | --- | --- | --- | --- | --- | --- | --- | --- | --- | --- | --- | --- | --- | --- |
